# Establishment of Stably Transfected Cells Constitutively Expressing the Full-Length and Truncated Antigenic Proteins of Two Genetically Distinct Mink Astroviruses

**DOI:** 10.1371/journal.pone.0082978

**Published:** 2013-12-23

**Authors:** Mehdi R. M. Bidokhti, Karin Ullman, Trine H. Jensen, Mariann Chriél, Amin Mottahedin, Muhammad Munir, Anna Maria Andersson, Olivier Detournay, Anne Sofie Hammer, Claudia Baule

**Affiliations:** 1 Joint R&D Division of Virology, Department of Virology, Immunobiology and Parasitology, The National Veterinary Institute (SVA), Uppsala, Sweden; 2 Division of Veterinary Diagnostics and Research, National Veterinary Institute, Technical University of Denmark, Copenhagen, Denmark; 3 Department of Biomedical Sciences and Veterinary Public Health, Division of Virology, Swedish University of Agricultural Sciences, Uppsala, Sweden; 4 The National Veterinary Institute, Department of Animal Health and Antimicrobial Resistance, Uppsala, Sweden; Instituto Butantan, Brazil

## Abstract

Astroviruses are becoming a growing concern in veterinary and public health. To date there are no registered vaccines against astrovirus-induced disease, mostly due to the difficulty to cultivate astroviruses to high titer for vaccine development using conventional techniques. As means to circumvent this drawback, we have developed stably transfected mink fetal cells and BHK21 cells constitutively expressing the full-length and truncated capsid proteins of two distinct genotypes of mink astrovirus. Protein expression in these stably transfected cells was demonstrated by strong signals as evaluated by in-situ PLA and IFA, and confirmed by Western blotting. The recombinant full-length and truncated proteins induced a high level of antibodies in mink, evaluated by ELISA, demonstrating their immunogenicity. In a challenge experiment in mink, a reduction in presentation clinical signs and virus shedding was observed in mink kits born from immunized females. The gene integration and protein expression were sustained through cell passage, showing that the used approach is robust and reliable for expression of functional capsid proteins for vaccine and diagnostic applications.

## Introduction

Astroviruses are non-enveloped viruses belonging to the *Astroviridae* family [1]. Members of this family infect the gastrointestinal tract of mammals and birds and are responsible for a large proportion of non-bacterial diarrhea, particularly in infants and elderly people, and in newborn of a wide range of animal species [2], [3], [4], [5], [6], [7], [8], [9], [10], [11], [12]. In turkeys and ducks they are further associated with kidney [13] and liver infections [14]. Infections in humans are widespread, with antibodies, for example, for HAstV-1 found in more than 90% of the population [2].

The genome of astroviruses is 6.4–7.3 kb in size, and is positive-sense, single-stranded polyadenylated RNA. It contains three open reading frames (ORFs), flanked by 5′ and 3′ untranslated regions [15]. ORF1a encodes non-structural proteins, ORF1b codes for the replicase, and they are translated into a polyprotein through a ribosomal frameshift mechanism [15]. This polyprotein is a precursor of the non-structural proteins that are generated from post-translational processing by viral encoded and cellular proteases [16], [17]. ORF2 at the end of the genome is transcribed from a single sub-genomic RNA of about 2.4 Kb, and translated into the capsid protein [18]. The size of the ORF2 product varies from 671–816 amino acids in different host species and astrovirus strains [19], and has a molecular mass of approximately 72 to 90 kDa [6], [15], [20].

Genetic analysis of ORF2 of astroviruses found in man and in different animal species has shown extensive variation between and within species [19], [21], [22], [23], [24]. Recently, human astroviruses found in fecal samples from cases of acute gastroenteritis in children were shown to be genetically related to the mink and ovine astroviruses [25], [26], [27]. In addition, an astrovirus found in the frontal cortex of a boy suffering from agammaglobulinemia was shown to be genetically close to mink astrovirus [28]. Also, a close similarity was found between astroviruses from pigs with those from humans [5], [29]. These reports strongly implicate that astroviruses may be regarded as potential zoonotic agents, which raises public health concerns and highlights the global relevance of these pathogens.

The ORF2-encoded capsid protein of astroviruses is the surface viral protein and has been shown to carry the antigenic determinants of the virus, it induces neutralizing antibodies [30] and mediates interactions with the host [31]. Thus, the capsid protein has been targeted for expression for processing studies [30], [32], [33], assembly, and to produce antigenic proteins [34]. The full-length capsids of human astrovirus-1 (HAstV-1) and HAstV-2 have been expressed using either recombinant baculovirus expression system [35] or vaccinia virus expression system [36]. The protein expressed in these systems has been shown to assemble into virus-like particles (VLPs).

Mink astrovirus (MiAstV) has been described as a causative agent of pre-weaning diarrhea syndrome in young mink kits [3]. The syndrome, referred to as “sticky”, “greasy”, or “wet” kits, is characterized by diarrhea and excessive secretion from cervical apocrine glands in mink kits, usually at the age of 1–4 weeks [3], [37]. Post mortem examination of kits dying from this syndrome reveals a non-specific catarrhal enteritis with hydropic epithelial cell degeneration, infiltration of mononuclear cells in the villous propria, and hypersecretion of the apocrine neck glands [38].

In a previous evaluation, a full-length capsid protein of mink astrovirus expressed and purified from *E. coli* was shown to induce high level of antibodies in immunized mink (Hammer et al. unpublished data). However, challenge with astrovirus resulted only in partial protection from symptoms of pre-weaning diarrhea and limited virus shedding. A baculovirus-expressed capsid protein of chicken astrovirus was evaluated to confer partial protection against the runting stunting syndrome [39]. There is a need to identify parts of the capsid protein combining immunogenicity with protective ability, features of an effective vaccine. In order to determine if truncated capsid proteins could have vaccine features, initially we constructed five recombinant clones from ORF2, based on predicted antigenicity plots of the mink astrovirus capsid protein: the full-length, two N-terminally truncated (CPΔN1, ORF2 nt 480–2325; CPΔN2, ORF2 nt 1152–2325) and two C-terminally truncated (CPΔC1, ORF2 nt 1–1863; CPΔC2, ORF2 nt 1–1200) forms of ORF2. On preliminary testing of the corresponding proteins in adult mink, only the full-length, the CPΔN1 the CPΔC1 proteins stimulated production of serum antibodies. Therefore the present study was conducted based on the constructs and proteins screened as potential vaccine candidates, in the path to develop a vaccine against mink astrovirus induced gastroenteritis. We have focused on development of a reliable source of recombinant capsid proteins through the establishment of stable transfected mink fetal and BHK-21 cells constitutively expressing ORF2 forms of two genetically distinct mink astroviruses, and performed primary challenge experiments with two of the proteins.

## Results

### Cloning of the Full-length, N- and C- Terminally Truncated ORF2

**Figure 1:**
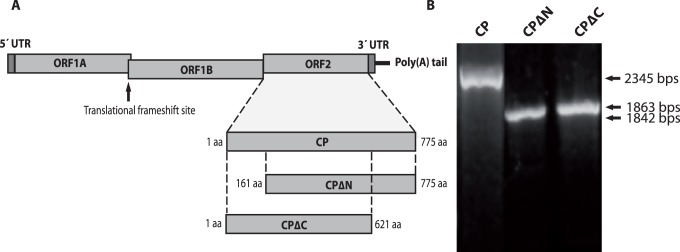
(A) Strategy for amplification of the full-length and truncated ORF2 of mink astrovirus. The full-length (CP), and truncated CPΔN and CPΔC fragments are represented. (B) Amplicons of the full-length (CP) and N (CPΔN), and C- terminally truncated (CPΔC) ORF2 of mink astrovirus generated with primers as described in Methods.

The strategy for amplification of the full-length, N- and C- terminally truncated ORF2 is depicted in Fig. 1A. Amplicons of the expected sizes were obtained by PCR (Fig. 1B) and cloned into the pDual-GC vector. Sequencing of the clones has confirmed the coding sequence for the capsid protein of mink astrovirus. The sequence of the ORF2 of mink astrovirus DK5790 was 99% homologous to the astrovirus sequence deposited in the GenBank with accession nr AY179509 and diverges by 49% from the sequence of a mink astrovirus found associated with the shaking mink syndrome (GenBank accession no. GU985458) (4). The sequence of ORF2 of mink astrovirus DK7627 is 47 and 44% divergent, respectively, to the astrovirus sequence of the enteric astrovirus deposited in the GenBank with accession no. AY179509, and to the sequence of the CNS disease inducing astrovirus described by Blomström *et al.* (2010) (GenBank accession no. GU985458) [40].

### Constitutive Expression of Astrovirus Capsid Proteins in Stably Transfected Cells

**Figure 2:**
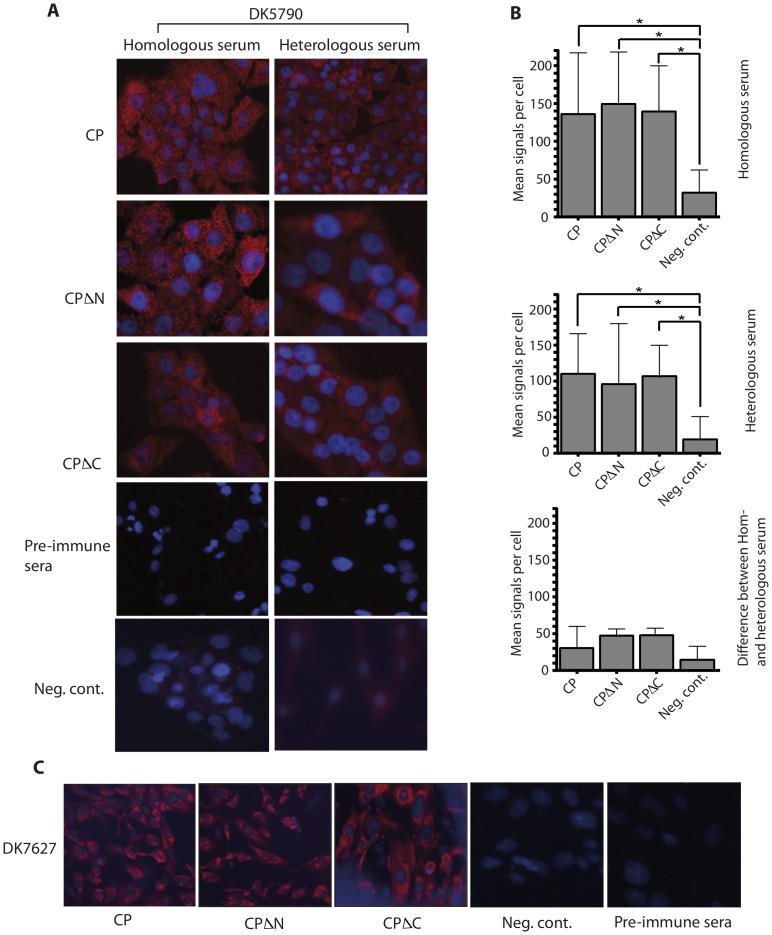
In situ-PLA and IFA in stable transfected cells. (A) The full-length (CP), N-terminally truncated (CPΔN) and C-terminally truncated (CPΔC) ORF2 constructs were transfected into mink fetal (MF) cells, subjected to G418 selection and clonal cells were thereafter tested for expression of the corresponding proteins by in-situ PLA as described in Methods. Sera to homologous (left panel) and heterologous (right panel) astrovirus were used, and thereafter cells were stained in an in-situ PLA. The controls were CP transfected and mock-transfected cells incubated with pre-immune sera or homologous/heterologous serum, respectively and stained as mentioned above. Signals of protein expression were present in the cytoplasm demonstrating stable transfection and constitutive expression of the indicated forms of the capsid protein in MF cells. (B) Quantitative expression of all three constructs for homologous (upper panel), heterologous (middle panel) antibodies and difference between homo- and heterologus (lower panel) is shown, as determined using Duolink ImageTool. (C) BHK21 cells transfected as before with DK7627 constructs were incubated with homologous serum and stained for IFA. Signals of protein expression were present in the cytoplasm demonstrating stable transfection and constitutive expression of the indicated forms of the capsid protein in BHK21 cells.

The three constructs from each astrovirus were successfully cloned and transfected into mink fetal and BHK-21 cells. The cells were subjected to G418 selection and the resistant clonal cells were selected and expanded. The cells were screened by in-situ PLA to evaluate expression of the cloned genes and to quantify specific signals for protein expression. Once determined that the signals are significantly different between cells transfected with ORF2 constructs and mock-transfected cells, IFA was used as a cheaper method to confirm constitutive expression in the cells. The transfected cells displayed signals for protein expression uniformly distributed in the cell cytoplasm as shown by PLA (Fig. 2A) and IFA (Fig. 2C) in different cell types. There were no differences in protein expression from different capsid constructs in mink fetal cells (Fig. 2A) and BHK-21 cells (Fig. 2C), evaluated by PLA and IFA, respectively.

Cells expressing full-length, CPΔN and CPΔC recombinant capsid of strain DK5790 reacted strongly with the antiserum of astrovirus genotype 1, i.e., homologous serum (Fig. 2A, left, top, middle and bottom panel, respectively), whereas reactivity to genotype 2 serum, i.e., heterologous serum was reduced (Fig. 2A, right, top, middle and bottom panel, respectively), though not significantly. Mock-transfected cells (negative control) and transfected cells incubated with pre-immune serum (shown for mink fetal cells transfected with the CP construct) did not show specific signals for expression of capisd proteins. Quantitation of the in-situ PLA signals showed a significant difference between signals in transfected and mock-transfected cells (negative control) (Fig. 2B).

### Expression Analysis of Recombinant Capsid Proteins

**Figure 3:**
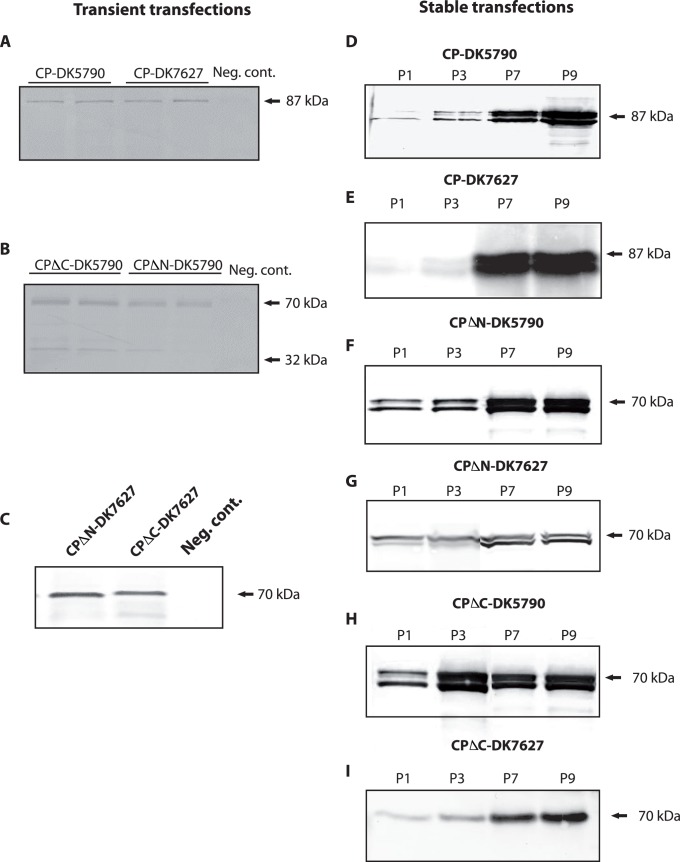
Expression of forms of the capsid protein of astrovirus in transient and in stable transfected mink fetal (MF) cells. The primary antibody was polyclonal serum for genotype 1 astrovirus, i.e. homologous to strain DK5790. Western blotting was performed as described in Methods. A) Transient expression of the full-length CP of astroviruses DK5790 and DK7627. (B) Transient expression of CPΔN and CPΔC proteins of DK5790. (C) Transient expression of CPΔN and CPΔC proteins of DK7627. (D, F, H) Expression of protein from MF cells stable transfected with CP, CPΔN, and CPΔC, respectively, of astrovirus DK5790. (E, G, I) Expression of protein from BHK21 cells stable transfected with CP, CPΔN, and CPΔC, respectively, of astrovirus DK7627.

**Figure 4:**
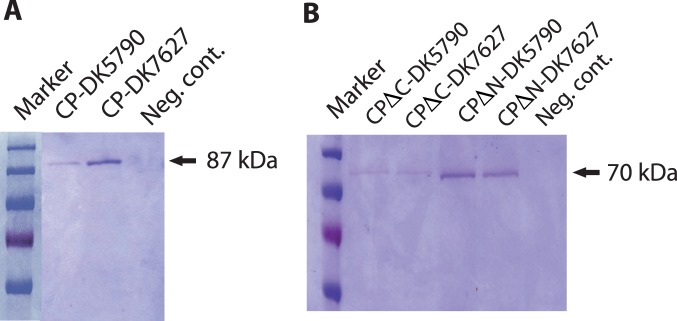
SDS-PAGE of purified proteins expressed from stable transfected mink fetal cells. The proteins were purified by nickel affinity chromatography as described in Methods. Ten microliters of protein were loaded in the gel, separated by electrophoresis and stained with Coomassie blue.

Protein extracts prepared from transiently and from stably transfected cells were analyzed for protein expression by Western blotting with antibodies directed to the astrovirus capsid protein. In extracts from transient transfections, a band was seen at the expected size for the full-length protein, approximately 87 kDa, for both genotypes (Fig. 3A). In transient transfections with constructs of the CPΔN and CPΔC constructs of DK5790, a band of approximately 70 kDa was present in the blots, with secondary band of approximately 29–32 kDa (Fig. 3B). Bands of approximately 70 kDa were seen in extracts of cells transfected with constructs CPΔN and CPΔC, respectively, of DK7627 (Fig. 3C). Proteins expressed in transiently transfected cells were in low amounts. In contrast, expression from stably transfected cells was higher and sustained through cell passage (Fig. 3D, CP), (Fig. 3F, CPΔN) and (Fig. 3H, CPΔC). The same pattern was seen in lysates of BHK21 cells stably transfected with constructs of astrovirus DK7627 (Fig. 3E, CP), (Fig. 3G, CPΔN) and (Fig. 3I, CPΔC). The protein yield varied between 500 µg and 1mg/ml, following purification from lysates of confluent cells grown in 175 cm^2^ surface flasks. An example of proteins purified from cell lysates is shown in the SDS-PAGE gel of Fig. 4.

### Integration of ORF2 Genes in the Transformed Cells

**Figure 5:**
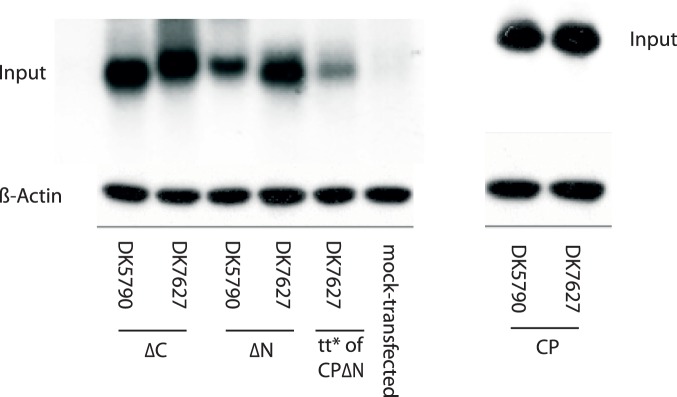
Southern blot for detection of gene integration in stable transfected mink fetal cells. DNA was isolated from cell extracts and 10 µg of each DNA were run on agarose electrophoresis, transferred to Hybond N membrane and hybridized with an astrovirus specific probe. Transiently transfected and non-transfected cells were included as controls. The signals were revealed by radiography scanning in a phosphorimager. Hybridization signals were detected in extracts of cells stable transfected with constructs CPΔC of DK5790 and DK7627, CPΔN of DK5790 and DK7627 (left blot) and CP of DK5790 and DK7627 (right blot). The signals were stronger in stable than in transiently transfected cells, as exemplified with transient transfection (tt) of CPΔN of DK7627.

In the Southern blot of DNA extracts from lysates of cells stable transfected with plasmids CP, CPΔN and CPΔC, signals from hybridization of specific astrovirus probe were detected, as shown for mink fetal cells in Fig. 5. The signals were stronger in stable than in transiently transfected cells. Since the DNA was not subject to restriction endonuclease digestion, integration was determined, but not a map showing the genomic location of the integrated sequences. This will be subject to further characterization of the expressing cells to analyse the patterns of integration and possible effects in protein expression.

### Immunogenicity of the Expressed Capsid Proteins in Mink

**Figure 6:**
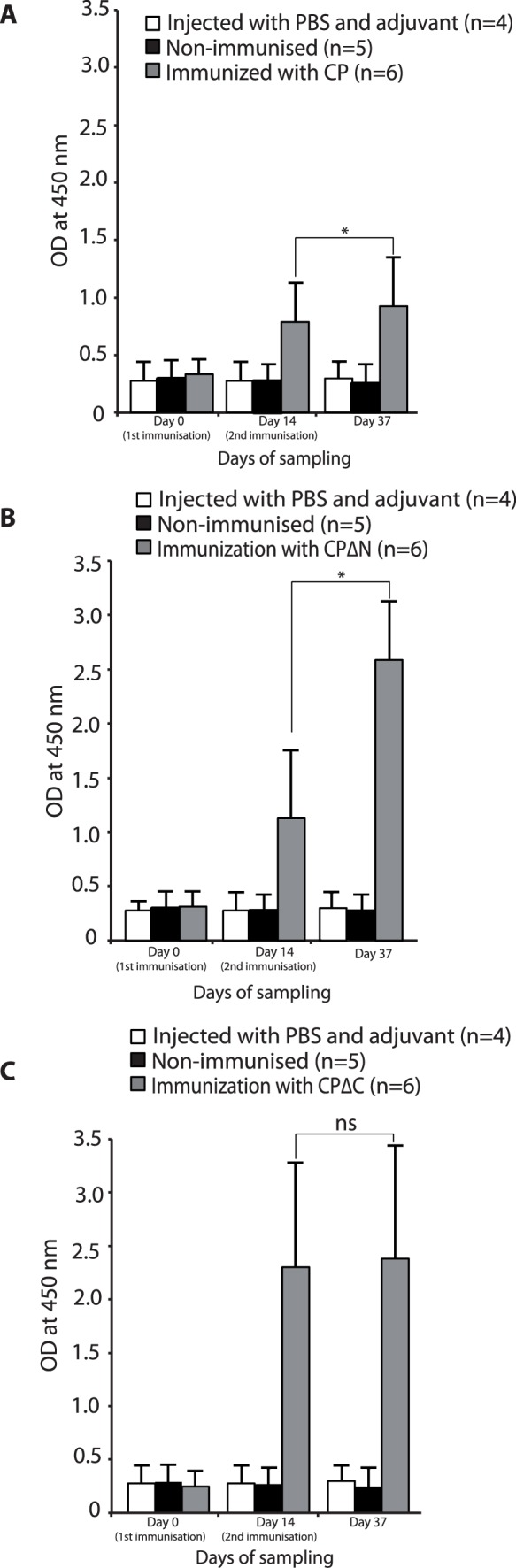
Antibodies to the mink astrovirus capsid proteins determined by ELISA. Adult mink were injected with proteins CP (A), CPΔN (B) and CPΔC (C) combined with Freund’s adjuvant, with two-week interval. Control mink received PBS plus Freund’s adjuvant injection on each occasion. The results of an indirect ELISA to determine antibodies in sera of the mink are presented as mean OD values. Asterisk show statistically significant difference between the levels of antibody at two time points (p<0.05).

Preliminary immunizations were performed by subcutaneous injection of six adult mink, three weeks apart with the CP, CPΔN and CPΔC proteins from astrovirus DK5790. Sera from animals were collected two weeks after each immunization and tested for antibodies in an ELISA. As illustrated in Fig. 6, the serum antibody to the capsid proteins showed a significant increase after the first injection in the immunized groups, compared to non-immunized group (p<0.005). The antibody level after the first injection of the CPΔC protein was significantly higher than that of CP and the CPΔN proteins (p<0.05) (day 14). Following the second immunization, the level of antibodies increased significantly (p<0.05) in mink injected with the CPΔN protein, while it remained unaltered in the groups of mink injected with the CP and the CPΔC proteins.

### Response to Challenge with Mink Astrovirus

**Figure 7:**
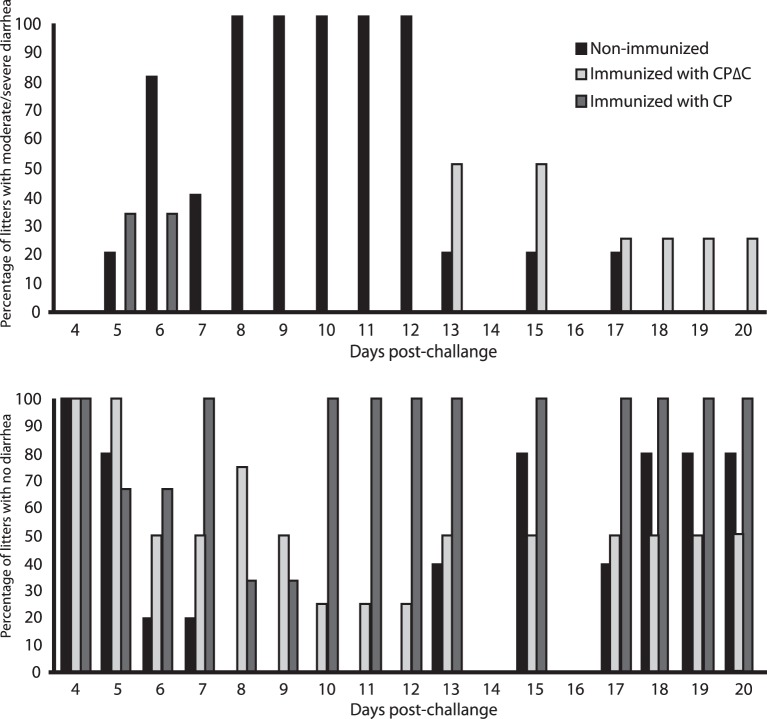
Scoring for diarrhea at different days post-challenge of mink kits. Mink kits born from mothers immunized with the CP and CPΔC proteins, and born from non-immunized females were observed for duration and severity of diarrhea, and categorized as with moderate/severe diarrhea, or with mild or no diarrhea. Higher percentage of litters with moderate/severe diarrhea in the critical period of 6 to 12 days post-challenge was seen in litters from non-vaccinated mothers, whereas they were reduced, and at a later stage in litters from CP and CPΔC immunized females (top panel). The bottom panel reflects the opposite for cases of mild or no diarrhea.

Astrovirus infection in mink primarily affects the kits, and the resulting disease, pre-weaning diarrhea syndrome, can only be reproduced in mink kits. Therefore, kits born from immunized female, and from non-immunized mink as controls, were challenged with astrovirus. Signs of diarrhea were reduced in days of presentation and severity in kits born from the CP and CPΔC immunized animals, by 70% and 50%, respectively (Fig. 7), while 100% of kits from non-immunized mothers had severe diarrhea lasting 5–7 days. In a previous challenge experiment, the reduction of severe diarrhea had been of 50% in kits from mothers immunized with a CP protein expressed in bacteria.

**Figure 8:**
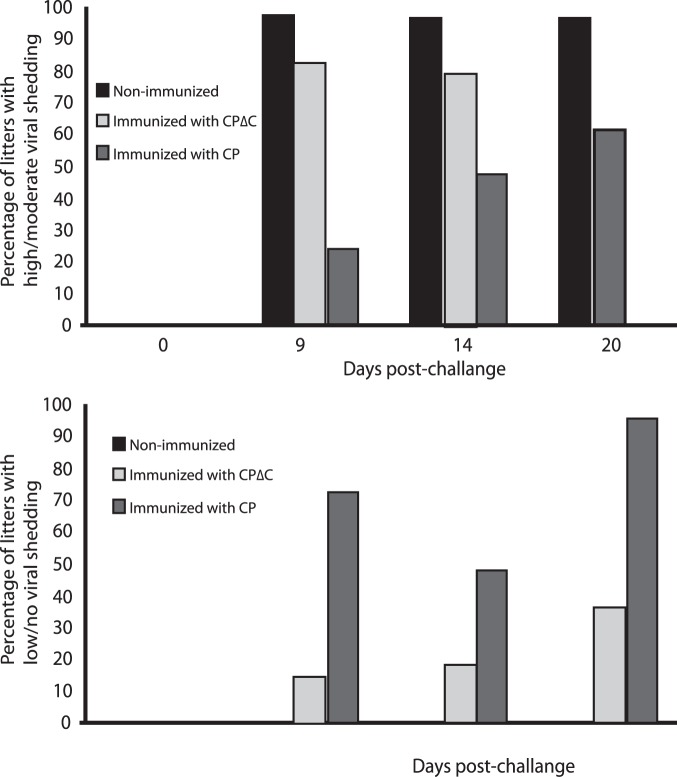
Virus shedding in fecal samples evaluated by real-time PCR. Samples collected from the litters at different days post-challenge with mink astrovirus were analyzed by real-time PCR and graded as high to moderate or low to negative for content of astrovirus as per copy number criteria described in Methods. The percentage of high/moderate virus shedders was higher in litters of non-immunized and CPΔC immunized mink.

Shedder mink were divided into three categories: high shedding, with astrovirus copy number more than 2*10^6^, moderate shedding with copy number between 2*10^3^ and 2*10^6^, and low shedding/negatives with copy number less than 2*10^3^ or no virus detection. The highest reduction in virus shedding (low or no virus shedding) was observed in kits from CP immunized mothers (75%), when virus shedding in kits from non-immunized challenged controls was high and in 100% of the litters during the critical period for presentation of diarrhea. Kits from mothers immunized with the CPΔC had moderate shedding detected in 50% of the litters, compared to controls (Fig. 8).

## Discussion

Astroviruses are difficult to culture *in vitro*, therefore production of vaccines based on live or inactivated virus has been hampered, as well as the use of serological tests that depend on growth of virus such as the neutralization assay. Molecular methods feature as an attractive approach to develop recombinant proteins for various purposes, including vaccines and systems to study the biology of astroviruses. The capsid protein encoded by ORF2 of astrovirus contains antigenic determinants [30] and mediates interactions with the host [31]. Genetic analysis of ORF2 gene of mink astroviruses found in fecal samples shows the existence of different variants of mink astrovirus (unpublished data). In addition, mink were recently found to be affected by a central nervous system disease caused by an astrovirus [40] that is genetically distinct by up to 47% to published sequences of mink astrovirus. Altogether, this shows a spectrum of genetic diversity and disease syndromes induced by mink astrovirus that needs to be taken into account for effective control of infections by means of vaccination.

In previous work, a recombinant full-length capsid protein of mink astrovirus produced in bacteria was evaluated for the ability to induce antibodies and to protect offspring of immunized pregnant mink upon challenge with astrovirus (Hammer *et al*. unpublished data). The results have shown a high level of antibodies induced by this protein, reduced clinical manifestations and reduced virus load in kits from immunized females compared to those of non-immunized controls. As a part of vaccine development studies, in the present work we have developed a system to improve expression of recombinant capsid proteins of astrovirus in order to carry out immunogenicity and protection studies. We have engineered mammalian cells to integrate the full-length and truncated forms of ORF2, encoding the capsid protein of mink astrovirus aiming at expression of proteins with the modifications that may be required for the protein’s immune function. The expression of cloned genes in mammalian cells allows post-translational modifications that do not occur in bacterial systems, therefore maintaining functional features of the expressed proteins [41].

The stable transfected cells were shown to constitutively express astrovirus proteins by immunofluorescence and in-situ PLA. The fact that the expressed proteins reacted well to homologous but weakly to heterologous sera imply that the sequence differences in the respective capsid proteins may determine antigenic changes. Therefore, understanding the immunogenicity of the capsid protein of different astroviruses and its implications is relevant to develop effective, broad protective subunit vaccines. The percentage of similarity between the two strains is low enough to define two different species of astrovirus than serotypes within species, as it has been suggested in the current ICTV taxonomy on astroviruses.

Western blotting analysis has revealed proteins of the expected size for the full-length, the N- and the C-terminally truncated capsid proteins, of approximately 87 and 70 kDa, respectively. In addition, secondary bands were seen in the blots of CP and CPΔN, which is consistent with the sizes of astrovirus cleavage products described upon infection in mammalian cells [34], [42]. It has been a common observation that capsid proteins from lysates of stable transfected cells show as a double band in the Western blots, but an explanation for this has not been found.

Expression of astrovirus ORF2 in vaccinia [36] and baculovirus [35] systems have been shown to result in the formation of VLPs. Despite several attempts we were unable to demonstrate VLP formation from the capsid proteins expressed in the mink fetal or BHK-21 cells, probably due to differences in preparation methods. Although VLP structures could not be demonstrated, the expressed proteins were shown to elicit an antibody response when injected in mink, indicating that they carry epitopes for immunogenicity. This shows that the capsid protein of mink astrovirus expressed in mammalian cells is immunogenic, as has been described for the capsid of other astroviruses expressed in baculovirus [35], [39].

Mammalian cells are frequently used as a host for expression of foreign genes for various functional studies, because they express proteins in stable biological and functional forms. Despite this, mammalian cells have not been extensively used to express recombinant proteins for vaccine purposes, contrary to their exploitation to grow and produce live attenuated or later inactivated viruses for vaccines. A common drawback is the low and unreliable protein expression from transiently transfected cells, as also seen in the present study. In this work we have used the approach of expressing the proteins from transfected cells to overcome limitations with yield and instability of expression. The stable transfected cells expressed proteins to yields of 500 µg/ml to 1 mg/ml. Moreover, the cloned genes were shown to be stable in the selected cells. There was no difference in protein expression in mink fetal and in BHK-21 cells, indicating that stably transfection could be flexible to different cell types.

The level of protein expression from heterologous genes introduced into mammalian cells depends upon multiple factors. These include DNA copy number, efficiency of transfection, mRNA processing, translational efficiency, protein processing and variations in codon usage between species. The use of codon-optimized genes may overcome the latter, improving levels of expression several fold from randomly optimized sequences [51]. Codon optimization has been reported to significantly increase protein expression in mammalian [51], [52], yeast [53], and bacterial cells [54]. For parvovirus B19 capsid genes, codon usage rather than promoter type and sequences of the 3′ UTR of the genes were shown determinant for increased expression in non-permissive cells [51]. The approach was not used in our studies, but is a strategy that would enhance protein expression, protein immunogenicity and reduce costs of scaling up. Different genes exhibit differences in efficiency of expression [41], thus, optimization conditions for expression of each gene are often required. Under the conditions used in our system, we have successfully integrated astrovirus genes and expressed the corresponding proteins from stably transfected cells. The system is amenable for scaling up by use of procedures for large scale tissue culture, for example in bioreactors, or in 3D cell culture systems, both for scaling up and for mimicking a suspension environment instead of growth on flat surface. For properly selected clonal cells it is not necessary to keep G418 selective pressure.

In the regime tested of immunization of females with recombinant proteins CP and CPΔC at gestation, followed by challenge of kits with a high virus doses, a reduction in duration and severity of clinical symptoms was observed in kits, at an average of 70% and 50%, respectively. The evaluation has shown protection of kits against severe diarrhea that is the clinical outcome of astrovirus infection in mink. Thus, the presented results support the feasibility of a capsid-based vaccine for protection against astrovirus disease in mink. Further trials will be performed to optimize the vaccination schedule.

It has been demonstrated that the capsid protein of human astrovirus type 1 blocks the classical [43] and lectin [44] activation pathways of the complement system, resulting in suppression of the downstream complement activation products and inflammatory mediators [43], [44], [45], [46]. The region involved in inhibition of complement activation has been mapped to span amino acids 79–139 [31], [44]. The CPΔN protein, where the truncation spans amino acids 1–160 presumably can overcome suppression of the immune system that has been described for the capsid protein. However, the CPΔC clone comprises the amino-terminal part of the capsid protein that is not processed and has been reported dispensable for VLPs assembly [47], [48]. Immunization of pregnant mink with this protein has shown to reduce severity of diarrhea in their born kits at challenge. The level of protection was similar to that conferred by a bacterially expressed protein in percentage of litters affected (50%) rather than in severity (the cases recorded were moderate). This appears to indicate either a limiting effect of the immunosuppressive region in the context of a truncated protein.

The molecular mechanism of astrovirus binding to host receptors has not been elucidated. However, it has been revealed that the amino acids that are lined up for receptor binding on the viral capsid protein are highly conserved [49], [50]. In line with this, human astroviruses show pairwise percentage identities of 47–57% in the capsid protein (in the P2 domain, from amino acid 415–646). Despite these facts, the two strains investigated in this study show remarkable differences in their amino acid sequences, which warrants further studies using predicted protein structures and crystallography to properly understand the biological relevance of these differences.

## Conclusions

In this work we developed mammalian cells constitutively expressing the full-length and truncated proteins of the mink astrovirus capsid protein for evaluation of their vaccine properties. The proteins were shown to be immunogenic in mink. The full-length and a truncated protein reduced the presentation and severity of clinical signs in challenged mink suggesting the potential a for subunit vaccines that can elicit an immune response without the risk associated with the use of live or attenuated virus.

## Materials and Methods

### Cells and Samples

Mink fetal cells (MF), passage 9, and baby hamster kidney (BHK-21) cells, passage 17, were grown in DMEM and EMEM medium, respectively, supplemented with 100 U/ml of penicillin and 100 µg/ml of streptomycin, 10% horse serum and 2 mM of L-glutamine, at 37°C in a 5% CO_2_ incubator. Two fecal samples (DK5790 and DK7627) from mink kits with diarrhea, identified as astrovirus positive by electron microscopy and PCR, and with distinct sequences in the ORF2 region, were used as source of virus. Virus DK5790 was homologous in the capsid region to the sequence of mink astrovirus with GenBank accession number AY179509 [6], while ORF2 of DK7627 had a nucleotide sequence distance of 47% to the AY179509 sequence.

### PCR Amplification of the Full-length and Truncated ORF2

Total RNA was extracted using proteinase K digestion and phenol/chloroform extraction protocol, followed by ethanol precipitation in presence of 0.1 M sodium acetate. Precipitated RNA was recovered by centrifugation at 13000 rpm, and subsequently washed in 70% ethanol. The RNA pellet was air-dried and resuspended in 20 µl of RNAse free water. Double stranded-cDNA (ds-cDNA) was prepared using Superscript II reverse transcriptase and an antisense primer complementary to the 3′ end of the genome for 1^st^ strand synthesis. Thereafter, the second strand reaction was completed by adding 30 µl of 5×2^nd^ strand buffer, 2 µl of dNTPs (10 mM), 1 µl of *E. coli* DNA ligase (10 U/µl), 4 µl of *E. coli* DNA polymerase (10 U/µl), 1 µl of *E. coli* RNAse H (2 U/µl) and distilled water to a total volume of 150 µl. All the reagents used were purchased from Invitrogen. The reactions were incubated at 16°C for 2 hours, then 2 µl of T4 DNA polymerase was added and incubation was continued for 5 min. The ds-cDNA was purified with phenol/chloroform and precipitated in ethanol. After pelleting by centrifugation and washing, the cDNA was used as template for the PCRs.

The full-length (CP), the N-terminus truncated (CPΔN) and the C-terminus truncated (CPΔC) fragments of ORF2 were amplified using *Pfu* Ultra DNA polymerase (Stratagene) and the primers listed in Table 1, following the manufacturer’s instructions. Briefly, a 50 µl reaction containing 5 µl of 10×Pfu Ultra buffer, 1 µl of 10 mM DNTPs, 1 µl of each primer (10 mM), 2.5 U of *Pfu* Ultra DNA polymerase, 1 µl of ds-cDNA and 40 µl of ddH_2_O was amplified using a cycling profile of 2 min of denaturation at 95°C, followed by 35 cycles of 95°C for 30 s, 55°C for 30 s, and 72°C for 4 min. In the last five cycles of amplification of the CP and the CPΔC fragments of strain DK5790, 1 µl of dm^5^CTP (Fermentas) was added to protect an Eam1104I recognition site present at position 125–131 of ORF2. A final extension for 7 min at 72°C was added. The PCR products were separated on 1% agarose gels and the DNA bands were visualized with ethidium bromide staining, under UV light. The PCR products were purified using the Wizard® SV Gel and PCR Clean-Up System (Promega).

**Table 1:** Primer pairs used for amplification of the full-length (CP), the N-terminus truncated (CPΔN) and the C-terminus truncated (CPΔC) fragments of ORF2 of mink astrovirus strains DK5790 and DK7627.

ORF2 geneFragment	Primerdesignation	Sequence (5′ to 3′)	Position in genomeof AY179509	ORF2 product size (bp)
CP of strains DK5790and DK7627	SMC1FSMC2R	*TAC TCT TCA* ATG GCG TCC GCC AAT CAG *TAC TCT TCG* AAG GTT CTT TGA GGA AAT TGC	4175–41926499–6482	23252334
CPΔN of strain DK5790	SMC3FSMC2R	*TAC TCT TCG* ATG TCA TTG AAT TTG ACA *TAC TCT TCG* AAG GTT CTT TGA GGA AAT TGC	4655–46726499–6482	1842
CPΔC of strain DK5790	SMC1FSMC4R	*TAC TCT TCA* ATG GCG TCC GCC AAT CAG *TAC TCT TCT* AAG CAT AAA GGT GCC ACT ACG	4175–41926037–6020	1863
CPΔN of strain DK7627	SMC3FNSMC2R	*TAC TCT TCG* ATG ATC TCC CTT AAC CTC *TAC TCT TCG* AAG GTT CTT TGA GGA AAT TGC	NA6499–6482	1854
CPΔC of strain DK7627	SMC1FSMC4RN	*TAC TCT TCA* ATG GCG TCC GCC AAT CAG *TAC TCT TCT* AAG GTT GTA GAC AAG CAA GTA	4175–4192NA	1890

F indicates the forward and R the reverse primer. The recognition site for *Eam*1104I is in italics. The AAG codon for continuing translation of the protein as fusion to the c-Myc tag is underlined (in SMC2R, SMC4R and SMC4RN). NA – not applicable.

### Cloning of the Full-length and Truncated ORF2

The primers used for the PCR carry a recognition site for the *Eam*1104 restriction enzyme to facilitate cloning. The pDual-GC expression plasmid (Stratagene) was selected due to the versatility of allowing expression both in bacterial and in mammalian systems. In addition, the vector contains His and c-Myc tags to allow detection and affinity purification of the expressed proteins. The PCR products generated for ORF2 of mink astrovirus and the vector were cleaved with Eam1104I restriction enzyme (Fermentas). The vector was dephosphorylated by treatment with shrimp alkaline phosphatase. Thereafter the vector and the PCR products were ligated with T4 DNA ligase. The ligation reaction was used to transform XL1-Blue MRF cells by electroporation, according to the manufacturer’s instructions. The bacteria were spread on LB agar plates containing kanamycin and incubated at 37°C overnight. Screening for recombinants was done by PCR. The positive colonies were propagated and used to purify the plasmid DNA, using Wizard® Plus SV Miniprep kit (Promega). The clones were confirmed by sequencing both strands using the Big Dye Terminator DNA sequencing kit (Applied Biosystems).

### Transfection, Selection and Propagation of Clonal Cells

The plasmid pDual-GC carries a neomycin resistance gene that confers resistance of successfully transfected cells, under Geneticin treatment (G418) (Invitrogen). BHK-21 or mink fetal (MF) cells were transfected with recombinant clones of full-length and truncated fragments using lipofectamine™2000 (Invitrogen). For transfection, the cells (1.5×10^5^ cells/ml) were grown in 6-well plates and subsequently transfected with 1 µg of the corresponding expression clones. The cells were then subcultured in medium containing 250–500 µg/ml of Geneticin in order to select cells resistant to neomycin. When clonal cells had been established, they were checked for integration of the ORF2 genes by PCR. This procedure was repeated upon cell passaging, to evaluate the stability of the inserted genes. Cells showing a positive PCR signal for gene integration were then propagated and analysed for expression of the proteins. Cells found to express protein were further propagated and then frozen at −134°C for future use.

### Immunofluorescence Analysis and in-situ PLA on Stably Transfected Cells

Polyclonal antibody as serum recognizing the astrovirus capsid protein has been raised by immunization of mink with purified full-length capsid protein expressed in *E. coli*. Positive reaction with the antibody and negative reaction with the pre-immune mink serum in Western blot using the protein as antigen was used to evaluate the antibody. The stably transfected cells were grown on 8-well chamber slides and when confluent they were fixed with 4% paraformaldehyde for 30 minutes. For immunofluorescence, the cells were permeabilized by incubation with 0.1% of Triton X-100 for 10 minutes, and then incubated with polyclonal antiserum for mink astrovirus, diluted 1∶100 and with pre-immune serum. The primary antibody was removed and the cells were washed three times with Tris-buffered saline (TBS) (20 mM Tris-HCl pH7.4; 150 mM NaCl) containing 10 mM glycine. A mouse anti-mustelid antibody diluted 1∶100 was added and incubated at 37°C for 60 minutes. The cells were washed and incubated as mentioned before with a donkey anti-mouse antibody labeled with Cy3 (Jackson Laboratories) as revealing antibody. The nuclei were stained by adding Hoechst 33342 (Molecular Probes) for contrast. The cells were washed as before, mounted and observed in a fluorescence microscope.

For in situ-proximity ligation assay (PLA), the cells were permeabilized as before and blocking was done by incubation with TBS containing 10% goat serum, at 37°C for 60 minutes. As primary antibody, homologous or heterologous polyclonal serum, and the pre-immune serum was diluted 1∶100 in TBS containing 5% whole goat serum, added to cells and incubated 37°C for 60 minutes. The primary antibody was removed and the cells were washed three times with TBS containing 0.1% Tween 20 (TBS-T). Then cells were incubated as before with a mouse anti-mustelid IgG diluted 1∶100. After washing, probing was done with PLA probes anti-mouse plus and anti-mouse minus. Ligation, amplification, detection, and washings in between steps were performed as recommended by the manufacturer (Olink Biosciences, Uppsala, Sweden, www.olink.com). The images were captured in a fluorescence microscope (Nikon Eclipse 2000) using corresponding filters for Texas Red (point-like PLA signals) and DAPI (nuclear) staining, with a Coolpix camera and NIS elements software. The Duolink® Image Tool was used to quantify the expression of each protein in the PLA. The total number of point-like PLA signals (single protein) and the total number of nuclei (average signal count) were counted after optimizing nucleus size, blob size, blob intensity and cytoplasm radius. Statistical analysis was performed using unpaired two-tailed Student t-test. Statistical significance was taken when p<0.05.

### SDS-PAGE and Western Blot Analysis of Expressed Proteins

The transfected cells were washed with phosphate-buffered saline and lysed in NP40 lysis buffer (20 mM Tris-HCl [pH 7.6], 150 mM NaCl, 5 mM EDTA, 1% NP-40, 0.5% sodium deoxycholate, and 0.1% sodium duodecyl sulphate) containing a complete protease inhibitor cocktail (Boehringer-Mannheim, Mannheim). The cell extracts were centrifuged at full speed in a refrigerated Eppendorff centrifuge for 10 minutes. Then, the pellet and supernatant were mixed respectively with 1× and 4×SDS sample buffer and separated on 12% SDS-PAGE gels. The proteins were then transferred to a PVDV membrane and analyzed by Western blotting by incubating successively with a polyclonal homologous antiserum against mink astrovirus as primary antibody, a mouse anti-mustelid IgG (dilution 1∶100) and a goat anti-mouse IgG conjugated to horseradish peroxidase (dilution 1∶2000). The incubations were done for 1 hour each step, with three times 10 minutes washes in PBS-Tween in between. Development was done using diamino-benzydine (DAB).

### Stability of Integrated ORF2 Genes in Transformed Cells

Integration of ORF2 sequences was investigated by Southern blot analysis. DNA was isolated from cell extracts by proteinase K digestion followed by phenol-chloroform extraction. The DNA was separated by electrophoresis in 0.8% agarose gel and subsequently transferred to Hybond N membranes by capillary transfer. The membranes were cross-linked, pre-hybridized, and hybridized with a probe, a 1250 astrovirus bp PCR product labeled with [32P]-dATP using a random labeling kit (Boehringer Mannheim). Following consecutive stringency washes with 2x SSC and 0.5 SSC buffers, the signals were visualized by radiography scanning in a BioRad FX phosphorimager.

### Protein Purification

Following confirmation by Western blotting, the proteins were purified by nickel affinity chromatography using HistTrap™columns (GE Healthcare, Uppsala, Sweden). Typically, cells were lysed on ice with phosphate buffer containing 20 mM immidazole, 1% v/v of Triton X-100, 5 µg/ml of DNAse I and RNase A. The lysates were equilibrated in phosphate buffer, pH 7.4 containing 20 mM immidazole as binding buffer, then run through the affinity columns. The columns were subject to successive washes to remove unbound material. The proteins were eluted in phosphate buffer containing 500 mM immidazole, and run through PD10 columns or dialyzed against PBS for buffer exchange. The purity of the proteins was evaluated by SDS-PAGE and the protein concentration was determined using the Pierce® BCA protein assay kit (Thermo Scientific, Waltham, MA, USA).

### ELISA for Detection of Antibodies to Mink Astrovirus

The antigen used in the ELISA is purified full-length capsid protein of mink astrovirus expressed in *E coli*. To detect antibodies specific to the capsid protein in mink sera, high-binding ELISA plates, MaxiSorp (Nunc, Roskilde, Denmark) were coated overnight at 4°C with 100 ng/well of protein, diluted in carbonate buffer (pH 9.6, SIGMA). The plates were washed three times with PBS-T (PBS with 0.05% Tween 20) and blocked with 100 µl of blocking buffer (PBS-T containing 5% of skimmed milk) for 1 hour. After washing, 100 µl serum sera diluted 1∶100 in blocking buffer were added and the plates were incubated at 37°C for 1 hour. A horseradish peroxidase-labeled mouse anti-mustelid IgG secondary antibody diluted 1∶1600 in blocking buffer was added after washing and the plates incubated at 37°C for 1 hour. Following washing and 10 minutes incubation with substrate solution (tetramethylbenzidine) the reactions were stopped by adding 50 µl of H_2_SO_4_. The optical density (OD) was measured at 450 nm with an ELISA microplate reader. The mean OD for the antigen-negative wells was subtracted from each result and the OD for each sample was corrected to a positive positive serum with the limit values of 1.5 to 2.0 OD in each run. Serum samples from pre-immune mink were used as a negative control with an OD-value of <0.2.

### Immunogenicity Trial with the CP, CPΔN and CPΔC Proteins in Mink

To evaluate immunogenicity, recombinant proteins CP, CPΔN and CPΔC of strain DK5790 were tested in mink. Adult female wild type mink were purchased from an Aleutian mink disease virus free farm without previously reported problems of astrovirus infection or other mink pathogens. Fecal samples were tested for astrovirus by real-time PCR and the sera for antibodies to mink astrovirus by ELISA, with negative results. The mink were divided into groups: one was injected with PBS and adjuvant (n = 4), one injected with the full-length CP (n = 6), one injected with the CPΔN protein (n = 6), and one injected with the CPΔC protein (n = 6). The injections were done subcutaneously with 100 µg of each purified recombinant astrovirus capsid protein in combination with Freund’s complete adjuvant. The immunization was repeated two weeks after the first immunization with the same amount of protein in Freund’s incomplete adjuvant. Blood samples were collected prior to immunization and three weeks after each immunization, and analyzed by ELISA.

### Challenge Trial with the CP and CPΔC Proteins in Mink

Adult female wild type mink were purchased from a farm without previously reported problems of astrovirus infection or other mink pathogens. The mink were tested for astrovirus and for antibodies to mink astrovirus, with negative results. Thereafter they were divided into groups of six mink each and immunised as follows: two groups were each injected subcutaneously with 100 µg of proteins CP and CPΔN and adjuvant mix; and one group consisted of non-immunized mink. The immunization was repeated three weeks after. The kits born from these females were exposed orally to a high dose of challenge astrovirus (10 million virus copies per millilitre). Clinical symptoms were recorded in the litters on a daily basis. Fecal samples were collected at different days post-challenge throughout the observation period and tested in a quantitative real-time PCR for determination of virus shedding after challenge. A standard curve was created based on a reference strain from experimentally infected mink, diluted 10-fold dilutions. The virus copy number was calculated and assigned to each dilution.

All animal experiments were conducted at the National Veterinary Institute, Technical University of Denmark in accordance with both institutional and national guidelines (Danish Animal Experiments Inspectorate, Permit Number: 2012-15-2934-00524). The experiments were approved by the Danish Animal Care and Ethics Committee, Denmark.
